# I don't think general practice should be the front line: Experiences of general practitioners working with refugees in South Australia

**DOI:** 10.1186/1743-8462-5-20

**Published:** 2008-08-08

**Authors:** David R Johnson, Anna M Ziersch, Teresa Burgess

**Affiliations:** 1 Discipline of General Practice, University of Adelaide, SA, Australia; 2 Department of Public Health, Flinders University, SA, Australia

## Abstract

**Introduction:**

Many refugees arrive in Australia with complex health needs. In South Australia (SA), providing initial health care to refugees is the responsibility of General Practitioners (GPs) in private practice. Their capacity to perform this work effectively for current newly arrived refugees is uncertain. The aim of this study was to document the challenges faced by GPs in private practice in SA when providing initial care to refugees and to discuss the implications of this for policy relating to optimising health care services for refugees.

**Methods:**

Semi-structured interviews with twelve GPs in private practice and three Medical Directors of Divisions of General Practice. Using a template analysis approach the interviews were coded and analysed thematically.

**Results:**

Multiple challenges providing care to refugees were found including those related to: (1) refugee health issues; (2) the GP-refugee interaction; and (3) the structure of general practice. The Divisions also reported challenges assisting GPs to provide effective care related to a lack of funding and awareness of which GPs required support. Although respondents suggested a number of ways that GPs could be assisted to provide better initial care to refugees, strong support was voiced for the initial care of refugees to be provided via a specialist refugee health service.

**Conclusion:**

GPs in this study were under-resourced, at both an individual GP level as well as a structural level, to provide effective initial care for refugees. In SA, there are likely to be a number of challenges attempting to increase the capacity of GPs in private practice to provide initial care. An alternative model is for refugees with multiple and complex health care needs as well as those with significant resettlement challenges to receive initial health care via the existing specialist refugee health service in Adelaide.

## Background

Under its Humanitarian program Australia accepts approximately 13,000 refugees each year [1]. Most of these will have experienced torture and trauma, forced migration and family separation, commonly the result of prolonged war or civil conflict [2,3]. Many will have had limited or disrupted access to medical care and will have spent long periods in refugee camps in environments that are extremely unsafe, where sanitation is poor and where access to safe drinking water and a nutritional diet is limited [4]. As a result, refugees often arrive in Australia with a complex mix of physical and mental health problems many of which are rarely seen in Australia [5-7]. The recent focus of the Australian Humanitarian Program has been to resettle those who have endured protracted refugee situations and who have originated from regions of very low socio-economic development. This has seen a large increase in the number of African refugees arrivals over the past 10 years – from 16% of the total annual intake in 1998/99 [8] reaching a peak of 70% in 2004/05 [1]. SA, which receives approximately 10% of the intake, has seen a similar change in the pattern of refugee resettlement [9]. Evidence is emerging that current refugee arrivals experience significantly poorer health status in addition to even greater resettlement challenges [10-17].

Despite the potential for high levels of morbidity, refugees undergo only a limited health assessment prior to their arrival in Australia. For the majority this includes a medical examination, a Chest X-ray for those 11 years and over and an HIV test for those 15 years and over [18]. Although more recently some refugees have received an additional health check a few days before departure, this is primarily to assess their 'fitness to fly' and a number of high prevalence infectious diseases and nutritional deficiencies are not included in this assessment [19]. As a result, refugees will often arrive with health conditions not previously identified [20] and there is a general consensus nationally[2,11,19,21] that all newly arrived refugees should undergo a voluntary comprehensive health assessment. Such an assessment would focus both on psychological as well as physical health needs in addition to providing information about illness prevention and health promotion activities and an introduction to the Australian health care system.

Until recently in SA, the State funded refugee health service along with two community health centres (CHCs) with specific refugee health expertise, performed comprehensive health assessments on a large proportion of newly arrived refugees to SA. With changes to the Department of Immigration and Citizenship (DIAC) funded Integrated Humanitarian Settlement Service (IHSS) contract in 2005, the responsibility of providing this initial care in SA was passed to General Practitioners (GPs) in private practice. Whilst this is the path taken in SA, each Australian state and territory has a different model for the provision of initial health care services with varying levels of involvement of specialist refugee and community health services [19], although GPs in private practice still provide a large proportion of this care. Recognising that GPs require extra assistance to do this work, the Federal Government introduced a new Medicare item number in May 2006 to better remunerate GPs who perform initial refugee health assessments.

There has been surprisingly little written documenting the experiences of GPs who provide initial care to refugees, both in Australia and overseas, including the challenges they might face and hence their capacity to do this work is uncertain. Some general review articles have been written by health practitioners based on their own experiences providing health care to refugees and challenges listed include those related to language, [3,22] the time consuming nature of the work [22,23], cultural differences [22,24] and the special health care needs of refugees [24]. Additional challenges found in a limited number of empirical studies overseas include GPs being unaware what previous screening or treatments refugees had undergone [25,26], that there were a lack of targeted services for refugees, [25] that refugees had greater heath care needs compared to non-refugees and that GPs lacked familiarity with the management of conditions unique to refugees [27].

There are limitations, however, in the applicability of these studies to the Australian context given differing national health systems and special service entitlements for refugees and the fact that it was unclear to what extent these studies related to the experiences of GPs providing care to refugees from similar backgrounds to those currently arriving in Australia. A review of the Australian literature found only one published study addressing this issue. In a report to the Victorian Department of Human Services, the Victorian Foundation for Survivors of Torture (VFST), drawing on their experiences as well as interviews with 19 GPs performing initial health assessments for newly arrived refugees in Melbourne, identified a number of similar challenges for GPs to those already listed [2]. Additional challenges included those related to the Australian Medicare fee-for-service system of remuneration which provided only a limited incentive for GPs to offer longer consultations or participate in 'extra consultation' activities often required when providing care to refugees. Challenges were also encountered relating to problems refugees experienced navigating the health system (eg. participating in follow-up GP care or attending referrals) and GPs faced difficulties sustaining their involvement because of the stressful nature of the work.

Whilst the VFST study provides valuable insight into the experiences of GPs in the Australian context, much of the initial health care for refugees in Victoria is performed by GPs working in State funded community health centres where they are more likely to be salaried and have better access to supports to assist them to manage patients with multiple and complex health needs. By contrast, in SA all newly arrived refugees are referred directly to GPs in private practice for initial health care and there has been no research to date assessing their experiences providing care to refugees in SA.

Given that current refugee arrivals are likely to carry a greater disease burden combined with the recent increased responsibility of GPs in private practice in SA to provide initial care, the aims of this study were: (i) to document the existence and nature of challenges for GPs who do this work in SA, (ii) to explore the ways in which these challenges could be reduced and (iii) to discuss the policy implications of this in relation to optimising the initial health care for refugees.

## Methods

### Design and participants

Given that the nature of this study was exploratory, a qualitative approach was taken in order to gain a deeper understanding of the challenges faced by GPs in private practice when providing care to refugees. Semi-structured interviews were conducted with 12 GPs providing care to refugees in private practice as well as the Medical Directors of three of the Divisions of General Practice in metropolitan Adelaide with high levels of current or proposed refugee settlement. The study was approved by the University of Adelaide Human Research Ethics Committee.

To recruit GPs, potential participants were identified via a database of GPs (held by the state funded refugee health service) who were either currently accepting or had accepted refugee referrals in the past. One of the authors (DJ) also used his personal knowledge of GPs known to provide care to refugees through his previous work at the SA specialist refugee health service and through formal and informal networks in the refugee health sector. Additional GPs were also identified following the Division interviews. An introductory letter with a fax-back reply was sent to 77 potential GP participants. After the initial mail out, six GPs agreed to participate. Follow-up phone calls were subsequently made to another ten GPs in order to recruit the remaining six GP participants. GPs were recruited from most regions of the Adelaide metropolitan area although there were none from the southern Adelaide region (resettlement of refugees to this region had only occurred relatively recently). The twelve GPs represented eight separate practices – two groups of three GPs were recruited from the same practices. Two thirds of participants had longstanding involvement in providing care to refugees whereas the remainder had become more recently involved with increasing numbers of African refugees resettling close to their practices. Whilst African refugees made up a large proportion of newly arrived refugees seen in the past twelve months, GP participants also reported providing care to large numbers of refugees from the Middle East as well as a small number of refugees from the Former Yugoslavia.

To recruit the Medical Directors of Divisions, five were contacted by email with two agreeing to participate. Follow-up telephone calls led to the recruitment of one further Division.

### Data collection

Given that a number of potential challenges were identified from the literature as well as there being much anecdotal evidence of challenges for GPs to do this work, a semi-structured interview format was chosen to examine these specifically whilst at the same time allowing any previously unknown challenges to emerge. Different interview schedules were used for the GPs and the Divisions respectively. Although the questions for each group focused on the broad aims of the study they addressed slightly different aspects of the issue. The GP interviews generally explored the challenges GPs face when working with refugees whilst the Division interviews focused on the current or potential role of Divisions to support GPs in private to work with refugees and well as the identification of potential structural impediments for GPs doing this work.

The interviews were conducted between April 2006 and July 2006. Each participant was interviewed once with interviews ranging from 40 to 70 minutes. Individual interviews were conducted with nine of the GP participants and a small group interview was conducted with the remaining three GPs. The three Divisions were each interviewed individually. The interviews were tape recorded and transcribed verbatim. In both the GP and Division interviews data saturation was reached.

### Analysis and reporting of results

A template analysis approach was adopted [28,29] where a coding template was developed which included *a priori *themes in addition to new themes identified from initial reading and analysis of the transcripts. Final thematic templates for both the GP and Division transcripts were agreed upon by the Project Team and then all data was coded according to these themes, with DJ undertaking the bulk of the coding. Two transcripts were also independently coded by the other members of the Project Team. Following this, comparisons were made and a consensus reached on how the remaining data was to be coded. Coding numbers were randomly assigned to protect the confidentiality of the participants where direct quotes were reported in the results.

## Results

GPs in this study reported a range of challenges when providing care to refugees. In many cases these challenges were explicitly linked to performing initial assessments whilst at other times GPs spoke of challenges in the broader context of providing care to refugees – but which can be assumed to be operating when providing initial care. The challenges fell into three main categories: (i) refugee health issues; (ii) GP/refugee interaction; and (iii) the structure of general practice. There was a great deal of overlap, however, between these categories and a very strong theme to emerge was not having enough time to do the work required which related to any issue that made a consultation with a refugee longer. Further, these challenges did not appear to be related to how long GPs had been providing care to refugees or to the intensity of their involvement other than that more experienced GPs had a greater awareness of available interpreter services. The Divisions also reported challenges assisting GPs to provide care relating to a limited awareness of refugee numbers settling in their divisions and which GPs needed extra support as well as a lack of specific Commonwealth funding to do this work. Finally, whilst participants suggested ways these challenges could be reduced, overall strong support was provided for initial health care to be provided via a specialist health service.

### Challenges for GPs

#### Refugee health issues

Challenges for GPs providing health care to refugees that related to refugee health issues included GP knowledge of previous health assessments, GP awareness of and experience managing health conditions unique to refugees, and the multiple and complex nature of refugee health conditions.

##### GP knowledge of previous health assessments

A number of GPs and Divisions expressed uncertainty regarding what health assessments refugees had received prior to arrival in Australia:

I don't know if there is some sort of system that they go through, or some sort of protocol that they, medically, have to go through before they are granted visas... (Dr 1)

There was also uncertainty regarding what health assessments were carried out after arrival in Australia with some GPs assuming that the MHS still performed this work. Uncertainty regarding previous assessments did not relate to how long GPs had been providing care to refugees.

For some GPs this resulted in confusion over their role in detecting and managing health conditions unique to refugees:

So we have got the clinical exotica; we have got very little understanding of what has happened to these people before and where the responsibility stops and starts for who should be following up all these things. (Dr 7)

##### GP awareness of and experience managing health conditions unique to refugees

Only one GP reported using guidelines to assist screening for exotic conditions. It was likely that many conditions unique to refugees were not being detected as indicated by one GP:

I haven't personally come across anything unusual that would be something that was quite rare... I'm sure I will. I'm sure I have probably missed heaps, too. Slipped through that I haven't seen or recognised. (Dr 1)

Concern was expressed, however, that refugee screening guidelines would just be another one of many such guidelines that GPs were expected to know about and follow.

Even if GPs were detecting conditions unique to refugees, there was concern that they did not necessarily have the experience to manage them:

I guess it is out of our comfort zone, because our medical experience doesn't include the exotic illnesses that they front up with... (Dr 7)

Again, these challenges were not dependent on how long GPs had been providing care to refugees as many of these challenges related to more recently arrived African refugees.

The expectation that GPs develop this expertise was also questioned as this was seen to compete with what was thought to be the important broader generalist role of general practice:

...we are supposed to be highly trained now in mental health and refugee health...when actually we are general practice. We are not specialty people...I think it is important that we stay that way, because you start going down into specialty areas too much and you start to miss the bigger picture... (Dr 1)

In terms of mental health problems, most GPs were aware of previous traumatic experiences of refugees and that combined with the stresses of resettlement meant that psychological problems often resulted. This also meant, however, that the time it took to build rapport and trust when seeing a refugee was far greater than with a non-refugee patient and this affected the ability to gather information about their background and past medical history.

##### The multiple and complex nature of refugee health conditions

Most GPs described providing care to refugees as demanding, with refugees more likely to have multiple and complex health needs:

...usually the refugees or migrants that I have seen have got multiple needs. It is usually not just one simple thing. (Dr 10)

This often meant the nature of the work was time consuming:

Often at the beginning there are so many issues to get through that I think it takes quite a number of long consultations before you really even have a clear idea about who this person is and what their experiences are. To get all the health issues on the table, I think, takes a really long time. (Dr 11)

#### GP-refugee interaction

A number of challenges relating to the interaction between GP and refugee were raised including issues related to culture and language as well as refugee knowledge of the Australian health care system.

##### Issues related to culture

A number of GPs observed that refugees often had a different understanding of disease causation when compared to a Western model:

...the way people behave around their health and their illness is very culturally determined. To try and understand what is going on, I can't just impose my framework, because the way they will express themselves is really different about what they are feeling. (Dr 11)

As a result, GPs reported having to spend much longer than normal explaining Western health concepts to refugees. This included screening activities such as Pap smears and organising referrals for mammograms, when giving a diagnosis of an illness such as Hepatitis B or when referring refugees for pathology tests. Other challenges attributed to cultural differences included uncertainty over cultural appropriateness of examination, gender related issues such as decision-making over birth control and gaining consent for invasive procedures:

...one woman came in and consented to a Pap-smear and thankfully, we managed to get around it that she actually had one before and was okay to do it, but I thought if she had never had one, how was I going to explain to this woman what I was going to do. (Dr 8)

Different naming practices also sometimes presented challenges in locating the correct patient file. Although GPs with longer standing involvement in refugee care may have been more aware of cultural differences, all GPs reported challenges related to this issue.

##### Issues related to language

A number of challenges relating to language resulted from the need to use interpreters. These included not being able to adequately provide explanations via an interpreter, difficulties dealing with mental health problems and the extra time required to both conduct a consultation with an interpreter as well as organise an interpreter when one was not pre-booked. Communication challenges were also experienced when contacting refugee patients for follow up of test results or to book an appointment. Although most GPs were aware of the need to use an interpreter when refugees were not fluent in English, those GPs with more recent involvement in refugee care were more likely to be unfamiliar with all the services offered by the Commonwealth Translating Interpreting Service (TIS), such as the doctor's priority phone line (where an interpreter can usually be made available on demand):

The times that I have needed it they have been – appointments have been booked well in advance. How do you book an interpreter when someone rings up at lunchtime and sees you two hours later for something that is minor or insignificant? (Dr 1)

One GP was confused about who paid for TIS believing that the practice was billed for an interpreting service when it was booked and the patient did not attend the appointment. A number of GPs also talked about difficulties contacting refugee patients for follow up of test results or to book an appointment:

Communication, when you know they can't speak English, so you can't phone them, and when you know that they are quite a mobile group of people, so that when you send a letter to their address, they might have moved on. (Dr 6)

##### Refugee knowledge of the Australian healthcare system

Almost all GPs mentioned difficulties resulting from refugees' lack of familiarity with the Australian health care system. This related to missed appointments, which meant no remuneration for GPs, or refugees arriving late:

That is a difficult problem with them... they will turn up really late for an appointment with no sort of seeming reference to the timeslot that they were given... it does sometimes make it difficult for us if we are then on the back foot for the rest of the session. (Dr 1)

GPs also reported that refugees' lack of understanding of the Australian health system resulted in challenges for GPs when they were referring refugees to other agencies or specialists and also when writing prescriptions. As a result GPs reported spending more time providing explanations about how the health system worked:

I take more time with them...Because the health care system here is much different... I try to make it easier for them to understand the system and how it works here. (Dr 5)

#### Structure of general practice

A number of challenges relating to the structure of general practice were identified including GP workforce shortages, a lack of organised referral pathways for refugees to general practice as well as a lack of remuneration and infrastructure support required to perform initial assessments.

##### GP workforce shortages

As a result of the demand for GP services outstripping supply in some regions of Adelaide, providing appointments for any new patients, whether they were refugees or not, was often difficult. Three GPs in this study, with high loads of patients with complex health care needs including refugees, had closed their books to new patients and another GP described potential difficulties accepting new patients:

We are having trouble accepting new patients full stop... freely accepting new patients irrespective of whether they are a refugee or not, it is difficult to actually accommodate everybody. (Dr 1)

Further, as highlighted by one Division, with GP shortages most acute in socioeconomically disadvantaged areas, refugees were more likely to be affected given that they are often settled in areas where housing was cheaper:

The cheaper areas for housing [are] where the workforce is the worst, so you can end up in this vicious circle where the practices go "Ah, we are closing our books, we are just not seeing anyone new". (Div 2)

##### Referral systems

Overall GPs reported that there was usually no clear referral pathway for refugees to private general practice. This was perceived as a problem because it meant that GPs were not able to control the numbers of refugee patients they saw when there were limits to the amount of work they could do with patients with multiple and complex needs such as refugees:

... because it is primary care you are expected to just take everybody that walks through the door. That doesn't work... lots and lots of agencies would like to refer here. We have to somehow prevent ourselves from drowning. (Dr 11)

Complicating this was the fact that when a GP took on one refugee then it was most likely that the rest of the family would then come to see the same GP which could dramatically increase their caseload of patients with high health care needs. Related to this was a fear for one GP clinic of being inundated with refugees if their clinic was promoted as a formalised referral centre for refugees because of difficulties already meeting the needs of their current patient load. A situation where there was no system in place to manage referrals was described by one GP as 'a recipe for burnout'.

Not having a formal referral pathway to GPs also led to problems with the transfer of health information including results from pre-departure health checks and any health services refugees had accessed in Australia. It was also noted that poor transfer of health information could also result in duplication of services such as immunisation.

##### Remuneration

Half of the GPs identified remuneration as a challenge when working with refugees. This was because refugees were mostly bulk billed and many needed longer consultations which were felt to be inadequately remunerated under the current Medicare billing system:

...it's just not financially viable because, as we know, long complex consultations are not a way which assists you to run your practice in a way that is financially viable... (Dr 11)

Remuneration was also a challenge because of the fact that refugees often missed appointments, which meant no remuneration, and work with refugees often involved time consuming extra-consultation activities that could not be charged to Medicare:

Missed appointments are fairly common...So you miss the remuneration if they don't come. The time factor; the complexity of the consult is more than what is remunerated for the time involved, because your follow-up is often phone calls to various agencies or organising things, writing letters, becoming an advocate, coordinating allied health. Very little of that is remunerated... (Dr 10)

Despite the strong indication that remuneration was a challenge for GPs, the majority of GPs said that they were either not going to use or were unlikely to use the new Medicare item number for performing an initial health assessment on a refugee although there was moderate support for it in principle. This was either because of a lack of familiarity with already existing Enhanced Primary Care (EPC) item numbers or because of the high administrative burden associated with their use:

It is not something I am likely to use personally. I think it is a great idea ... but I am not very good at using the specific numbers ... and the EPC items and so on. I just don't have time to sort out all the paperwork for that sort of thing. (Dr 6)

Another GP was disappointed that the Divisions of General Practice had not produced a template to assist using the item number in the same way that they had with previous EPC item numbers. Finally, a number of GPs were critical that the item number did not go far enough in that it failed to recognise that the greater health care needs of refugees were ongoing.

##### Infrastructure supports to perform initial assessments

There was strong evidence provided by the majority of GPs in this study that they did not have the necessary infrastructure support, i.e. the systems and support staff, to perform initial refugee health assessments:

We are not well enough equipped. We are not resourced, we do not have the supporting background structure. (Dr 9)

It was particularly overwhelming for GPs when groups or families all came at once and they had not had a previous health assessment:

...we were having whole families of recent arrivals come to the surgery and need all their history taken; immunisations brought up-to-date and that was just overwhelming... We do have a practice nurse, but she is usually quite busy doing other things. That was too much for us to handle. (Dr 6)

The Divisions also believed that a lack of infrastructure support was a reason it would be difficult for GPs to perform initial health assessments and was a reason uptake of the new item number would be limited:

If you just basically say "Here is a new item number doctors" it won't be taken up, because it is going to be all too hard. From an infrastructure perspective most practices lack the infrastructure to really make this work. (Div 3)

### Challenges for Divisions assisting GPs

The Divisions identified a number of challenges in assisting GPs to provide care to refugees. They expressed concerns that they did not know how many refugees were being resettled as well as precisely where these resettlements were occurring within their Divisions. The Divisions reported also having limited awareness of which GPs in their Divisions were providing care to refugees and, as a result, were not able to determine which GPs might need extra support to do this work. One Division had surveyed GPs to assess this but most had been too busy to respond. Although a number of GPs had been recruited by the IHSS provider to offer care to newly arrived refugees, the Divisions expressed frustration that they did not know who these GPs were. They were also concerned that there was no collaboration with the IHSS provider which might avoid resettling refugees in areas where they might have difficulty accessing GP services:

... there is no point putting refugees in to an area where there are no GPs. There might be GPs there but they might not want to see refugees or they might have closed their books. We would have that intelligence; they would have no idea about that... (Div 1)

All Divisions mentioned a lack of funding as a major reason their ability to help GPs was limited. Because refugee health was not a priority area for the Commonwealth, Divisions received no direct funding for refugee health initiatives:

We are funded by the Department of Health and Ageing; we have got a whole lot of quality indicators that we have got to actually achieve in. Refugee health does not even appear in there. (Div 3)

The Divisions explained that it was also a lack of funding that limited their ability to assist GPs to utilise the new item number. Despite this, two Divisions had diverted core funding to better support GPs in the area of refugee health but no Divisions had funding for specific services or programs. One Division felt their case to argue for increased funding was limited given the lack of data related to refugee numbers settling in the Division and that there was no empirical evidence that GPs weren't coping even though it was recognised that GPs were too busy to answer surveys that might provide this data.

### Ways GPs could be better supported

#### Providing GPs with more resources

Despite GPs questioning how realistic it was for them to manage many of the exotic health conditions in newly refugees, there was some support for the provision of screening guidelines for use by GPs in private practice provided they were in a simple and readily accessible format – such as linked to the general practice software program Medical Director (MD).

GPs reported they could be assisted to overcome some of the challenges related to culture if they were provided with more background information about refugee groups either through the provision of information sheets or talks from different community members regarding different cultural practices. It was also suggested that these challenges could be reduced through better provision by settlement services of health information to refugees on arrival including information about common conditions in refugees such as hepatitis B, early intervention and illness prevention activities and better initial orientation to the Australian health care system. Many GPs also believed that settlement services could provide more assistance to refugees to attend appointments. Other ways of improving refugee navigation of the health system included a greater role for voluntary organisations, practice nurses or community health care workers who could be health advocates for refugees:

At times you feel like you're running around doing a lot of the work that could be partly done through either a practice nurse or another allied health worker or somebody who can, on the ground, advocate for that refugee individual. (Dr 10)

In relation to referral pathways of refugees to general practice, it was strongly stated by a number of GPs that this should involve a consent process which assessed the ability of a GP to take on the care of a new refugee or refugee family:

It is not a kind of fair system to plonk someone onto a practice. I think agencies should actually liaise with either one of the senior doctors or, if there is a nurse... so a referral can actually be properly organised and assessed as to whether the practice can take someone on. I think the ways it has happened in the past have been really unsatisfactory. (Dr 11)

Despite limited enthusiasm for the new Medicare item number, a small number of GPs were still interested in learning more about how to use it through their Division and there was also some support for the provision of a template by the Divisions which could also be incorporated into MD.

The Divisions suggested a number of ways they could assist GPs to perform initial assessments if they were provided with funding. This included improving the utilisation of the item number by educating practice nurses and developing a template as well as Division funded 'clinical attachments' at the state funded specialist refugee service to provide GPs with greater expertise in managing health conditions unique to refuges. It was also suggested that Division funded refugee health infrastructure grants could provide assistance to GPs to better set up their clinics to care for refugees via IT support and the provision of business cases for employing practice nurses. One Division, however, expressed uncertainty about the likelihood that GPs would be willing to build such systems into their practices if they were only seeing small numbers of refugees, particularly given that there was limited enthusiasm to build similar systems for high prevalence chronic diseases in the general population.

#### Providing initial refugee health care via a specialist service

Despite indicating ways they could be assisted, a number of GPs believed that the responsibility of providing initial care to refugees should not lie with GPs in private practice:

There should be a front line somewhere. I don't think general practice should be the front line. (Dr 8)

Instead, a system where refugees received an initial assessment via a specialist refugee or community health service was strongly supported by both GPs and the Divisions. One GP described the advantages of a community health service where a range of services could be provided to refugees in one location:

I think the community health service is the best stop for an initial assessment because of the complexity of the presentations, usually, and the need for accessing a lot of different services that is really beyond most private centre GPs to be able to do that adequately. If you have got access within one building, for example, to workers who can do some of the chasing up, some of the phoning and some of the coordination, then you are much more likely to give people a good service. (Dr 10)

A number of GPs indicated that they would be much happier to accept referrals of refugees if they had had an initial assessment where they could then focus on their more day-to-day health needs.

## Discussion

This study highlights the many and diverse challenges faced by GPs in private practice when providing health care to refugees in their initial resettlement period. These challenges and their policy implications are discussed below.

### Challenges performing initial assessments

The extent of the challenges faced by GPs providing initial care to refugees in this study is not surprising given that it is during their early resettlement period that refugees are most likely to experience multiple medical problems, many of an exotic nature, when language and cultural barriers are likely to be greatest and when refugee knowledge of the Australian health care system and general health literacy are likely to be most limited.

Whilst many of the challenges identified support previous research in this area, the most striking feature of this study is the strong evidence that GPs in private practice are not sufficiently resourced to provide initial care effectively to newly arrived refugees with multiple and complex health needs. For GPs in this study, the lack of resources existed both at an individual GP level, with GPs lacking comprehensive knowledge of the health conditions unique to refugees, as well as at a more structural health system level, where GPs lacked both the time and the infrastructure support to do this work effectively. Further, the lack of resources was not related to the length of time GPs had been providing care to refugees or the intensity of their involvement other than that GPs with less experience were less familiar with the use of interpreter services.

Given that the first point of contact with the Australian healthcare system for refugees currently arriving in SA is with a GP in private practice, there are a number of health consequences for refugees if GPs do not have the necessary resources to provide them with effective care in their initial resettlement period. These include GPs missing or inadequately treating physical and mental health problems unique to refugees, refugees not following through with treatments or referrals and refugees under engaging with illness prevention activities because of poor health literacy [30]. This can result in refugees experiencing a reduced health status compared with the non-refugee population as well as potentially greater costs to the health system because of later and more expensive treatments.

### Implications for policy

#### Barriers to building capacity of GPs to perform initial assessments

Although GPs mentioned a number of ways they could be assisted to provide more effective initial care to refugees, they also indicated that there would be major limitations attempting to build this capacity.

Firstly, GPs questioned the expectation that they develop the specific 'refugee health' expertise needed for performing initial assessments which competed with their role as 'generalists'. It is likely that, at best, developing the required expertise to perform initial assessments will only appeal to a small number of GPs.

Secondly, even those GPs who have an interest in doing this work may not want to identify themselves as a 'refugee doctor' for fears, as stated by one GP, that they will become inundated with referrals. GPs in this study indicated that there were limits to the amount of work they could do with refugee patients given the often multiple and complex needs on initial presentation. GPs, however, operate in a primary health care (PHC) system where they have little control over how patients are referred to them. Further, GPs performing initial health assessments are most likely to be the GPs who provide the ongoing care (more so now given that refugees are initially settled in more permanent accommodation). As a result, GPs providing initial health care can quickly end up with very high numbers of patients with multiple and complex health needs. A number of GPs in this study indicated that this had contributed to them closing their books to new patients. Refugee health service providers in Adelaide as well as interstate[2] have also experienced the difficulties sustaining GP involvement under these circumstances. To avoid overburdening a small number of GPs would mean, however, offering more general training to a large number of GPs. This is unlikely to be a cost effective approach and also, as evidenced by this study, developing the necessary expertise and building the practice systems required to provide effective initial health care to refugees will, at best, appeal only to a small number of GPs.

Thirdly, GPs indicated that providing initial care to refugees was time consuming but the fee-for-service structure of general practice combined with GP workforce shortages limited the time GPs could offer to refugees to manage their multiple and complex health needs – a problem shared with other groups who have greater health care needs [31]. Under these circumstances, GPs are unlikely to take on a role that will require them to offer a greater number of longer consultations. This could be one reason why the new Medicare item number received limited support from GPs in this study despite the fact that a lack of remuneration was an issue for a number of them. As suggested by the Divisions, this could also be an indication that the new item number, in its current form, does very little to address the resource problems described above. It is interesting to note that the initial uptake of the item number was greatest in Victoria [32] where a large number of refugees receive initial GP care in community health centres with the support of refugee health nurses which highlights the importance of providing adequate time and infrastructure support when doing this work [33]. A further limitation of the item number, also mentioned by GPs in this study, is that it does not take into account the fact that the greater initial health care needs often persist beyond the first visit with a GP.

#### The role of a specialist health service

An alternative to providing initial care to refugees in private general practice is for this to be provided in a specialist refugee service or community health setting. Such a service delivery model received strong support from participants in this study. Previous studies have similarly highlighted the central importance of community health services providing initial health care to patients with complex health needs, including refugees, as a result of better access to resources and infrastructure support [2,26,34].

It is acknowledged that there is not a one size fits all approach when determining which model, specialist or mainstream, best meets the special service needs of refugees in their initial resettlement period [35] and that receiving PHC via a specialist service may delay refugee engagement with local mainstream PHC services [2]. Where there is limited capacity, for mainstream services to provide for these special needs, and this study provides strong evidence that this is the case in private general practice in SA, there is a role for a specialist service to fill this service gap [35,36]. In SA, such a state funded service already exists, although it is not currently being utilised as the current settlement service provider has adopted a policy of connecting refugees directly with mainstream health services immediately on arrival to Adelaide. It makes sense to utilise the current refugee health expertise and resources of this state funded service to provide initial health care services to refugees, especially those with complex health needs and significant resettlement challenges. Whilst this is a centralised service, the highly centralised population distribution of SA in Adelaide, combined with the relatively small number of refugee arrivals, means that it is accessible for the majority of refugees in SA. Further, the ability to deliver refugee services in multiple community health locations, such as in Victoria, is limited in Adelaide because of a lack of medical presence at these sites. It is recognised, however, that a centralised specialist service is not well suited to larger Australian cities such as Sydney and Melbourne. Finally, if initial health assessments are provided by a specialist service, it is important that a clear, transparent and effective referral system to a nominated general practice is part of this process when initial health care needs have been met. Ongoing links between general practices and the specialist provider would also address a number of the other challenges identified by GPs and Divisions in this research.

### Study strengths and limitations

This study provided a unique and detailed insight into the experience of GPs providing health care to refugees. However, given the small number of participants in this study, these results cannot be generalised to all GPs in Adelaide or GPs in other locations. To do this, a larger quantitative study would be required. The low response rate from GPs could have meant that those GPs involved were more motivated to participate because of dissatisfaction with the current system of provision of initial health care to refugees. This low response rate, however, mirrors the experience of Divisions in this study and their difficulties getting GPs to participate in research and respond to surveys. Further, it is generally believed that at the time data was collected for this study there were a limited number of GPs (although the exact number is not available) providing initial care to refugees in SA. It is likely, therefore, that the views of GPs in this study not only provide a reasonably comprehensive summary of the challenges of providing initial care but are also the experience of most GPs doing this work in SA at the time. Whilst GPs interstate are likely to face many similar challenges providing initial care to refugees, it is beyond the scope of this paper to comment on how well resourced they are to provide effective care.

## Conclusion

This study provides evidence that, due to a range of challenges, GPs in private practice in SA are insufficiently resourced to provide initial health care effectively to refugees and that attempting to overcome these challenges would face a number of obstacles. Whilst further evidence is required to document the extent of these challenges in SA and how they might be best addressed, it makes sense for the existing state funded refugee health service to be involved in the delivery of initial PHC services to refugees, especially those with complex health needs and significant resettlement challenges.

## Competing interests

The authors declare that they have no competing interests.

## Authors' contributions

DJ conceptualised the study, conducted the literature review, undertook data collection and analysis, and drafted the paper. AZ and TB advised on all stages of the work including analysis of the data as well as reviewing and contributing to drafts of the paper. All authors read and approved the final manuscript.
